# Editorial

**Published:** 1902-11-15

**Authors:** 


### EDITORIAL.

THE publishers of the *Dental Cosmos* and *Dental Digist* announce that beginning with the January 1903 issue both of these journals may be secured for one dollar per year. We are glad to know that the character of the matter presented will not be cheapened with the price. It is an effort undoubtedly to secure a much wider reading of these journals; we see no reason why this move may not accomplish this object. It is in line with the policy pursued by the secular press, and since it seems to meet with success, there should be no question but that it will do likewise for the professional journalists. It will no doubt cut into the subscription of the smaller journals which print only half as much matter, but as these journals mostly represent local fields, the injury to them will not be great. It ought to do one thing which may more than compensate this seeming loss of professional dignity; and that is to increase the habit of reading the current professional literature by a larger number of dentists, many of whom now read no journal, or perhaps only one. A five dollar bill judiciously expended ought now to put in the hands of every dentist sufficient reading to keep him well informed in all departments of his profession. There is now only one American journal which charges more than one dollar per year subscription, this is the *International Dental Journal* owned and published by the profession, and edited by Dr. Trueman. We shall be curious to see if there is sufficient loyalty in this organization to sustain its principle of independence in spite of this action of the journals owned by trade houses.

The Central Jersey Dental Association at its last meeting was entertained by the firm of Johnson & Johnson, of New Brunswick, the manufacturers of sterilized cotton preparations for dental and medical purposes. A visit of inspection to the factory and a banquet plentiful as to quality and unique as to menu, with two interesting papers made the occasion one of profit as well as pleasure. It would be a good idea if the profession could be brought into more intelligent touch with the dealers and manufacturers. The manufacturer might receive helpful suggestion from the profession in this way, and the practitioner would have a more intelligent idea of how the many articles he is accustomed to use are produced, consequently he would be enabled to discriminate as to merit, and be more likely to avail himself of every facility he could use to advantage in the practice of his profession.

The students of the Dental Department of Washington University, of St. Louis—as well as the other departments of the University—are to have a fine club house near at hand, where meals can be had, and opportunities afforded for all manner of social and athletic recreation. This will no doubt be an attractive feature and if managed as Huston Hall at the University of Pennsylvania will be of great value in cultivating a college *esprit de corpse*.

